# Rare Earth Material for Hydrogen Gas Sensing: PtGd Alloy Thin Films as a Promising Frontier

**DOI:** 10.3390/nano14131098

**Published:** 2024-06-26

**Authors:** Necmettin Kilinc, Susana Cardoso, Mustafa Erkovan

**Affiliations:** 1 Department of Physics, Faculty of Science & Arts, Inonu University, 44280 Malatya, Türkiye; 2 Instituto de Engenharia de Sistemas E Computadores–Microsistemas e Nanotecnologias (INESC MN), 1000-029 Lisbon, Portugal; scardoso@inesc-mn.pt (S.C.); mustafa.erkovan@sivas.edu.tr (M.E.); 3 Department of Fundamental Sciences and Engineering, Sivas University of Science and Technology, 58000 Sivas, Türkiye

**Keywords:** sputtering, gas sensor, hydrogen sensor, platinum, platinum–gadolinium alloys, thin films

## Abstract

At the focus of our investigation lies the precision fabrication of ultrathin platinum–gadolinium (PtGd) alloy films, with the aim to use these films for resistive hydrogen gas sensing. The imperative for sensitive and selective sensors to harness hydrogen’s potential as an alternative energy source drives our work. Applying rare earth materials, we enhance the capabilities of hydrogen gas sensing applications. Our study pioneers PtGd alloy thin films for hydrogen gas sensing, addressing a gap in existing literature. Here, we demonstrate the functional characteristics of 2 nm thick Pt_x_Gd_100′x_ (x = 25, 50 and 75) alloy films, analyzing their hydrogen gas sensing properties, comprehensively examining the interplay between alloy composition, temperature fluctuation and hydrogen concentration. The effect of composition and structural properties on the sensing response were assessed using EDX and XPS. The films are tested at a temperature range between 25 °C and 150 °C with hydrogen gas concentrations ranging from 10 ppm to 5%. Hydrogen gas sensing mechanisms in PtGd alloy ultrathin films are explained by surface scattering. The unique combination of Pt and Gd offers promising characteristics for gas sensing applications, including high reactivity with hydrogen gas and tunable sensitivity based on the alloy composition.

## 1. Introduction

Hydrogen, a ubiquitous energy source throughout the universe, plays a crucial role in diverse sectors including chemical synthesis (e.g., petroleum refining, ammonia production, metal processing), fuel cells and propulsion systems for both terrestrial and extraterrestrial travel [1,2,3,4]. As part of contemporary environmental priorities, hydrogen has elevated its status as a clean, renewable and effective energy alternative. This significantly reduces greenhouse gas emissions associated with fossil fuel burning. Therefore, hydrogen has emerged as a fundamental energy source with the potential to reshape the future energy framework [1,2,3,4].

Hydrogen’s potent reducing properties across multiple elements and its ability to penetrate diverse materials require rigorous control in specific circumstances. Moreover, hydrogen is invisible and has a propensity for combustion and explosive reactions at concentrations exceeding 4% in air. Motivated by safety concerns, hydrogen sensors have spread and are critical in preventing hazardous air mixtures and potential explosions [1,5,6,7]. These sensors are categorized into nine distinct categories based on their operational mechanisms as follows: electrochemical, catalytic, resistive (semiconductor and metallic), work function–dependent, acoustic, mechanical, thermal conductivity, optical and magnetic [5,7,8,9,10,11,12].

In the class of resistive metallic sensors, these devices often incorporate sensitive layers of palladium (Pd), platinum (Pt), or their alloys, marking significant advances in sensor technology. This sector has seen the development of various nanostructured Pt materials, including nonporous films [13,14], nanowires [15,16,17,18,19], thin films [20,21,22,23,24] and layered PtPd and PtTi thin films [25,26], as well as PtAu and PtPd nanoparticle layers [27,28], Pt-modified Pd nanowires [29], PtPd nanoparticle–polymer composites [30], Pt and PtRh nanosheets [31] and different alloy (PtPd, PtNi and PtCo) films [32,33,34]. These innovations underscore the versatility of Pt-based sensor technology, which allows for numerous structural adaptations to optimize hydrogen sensing.

Significant research has focused on the interaction between rare earth elements and hydrogen. This is driven by dramatic changes in their physical and structural properties at altered hydrogen levels [35]. The ability to alternate between reflective and transparent states with reversible hydrogenation and dehydrogenation is one of the most notable characteristics of rare earth–based hydrogen storage alloys [36,37,38,39]. Gadolinium (Gd), known for its high spin density and intrinsic magnetic moment due to its half-filled 4f orbitals, significantly influences spin configurations and electron distributions among adjacent metals—a critical aspect of hydrogen absorption, even under thin Pd coatings [40,41,42].

This article presents the preparation of PtGd alloy ultrathin films with different compositions for hydrogen gas sensing applications. The composition and structural characteristics of these films are evaluated using EDX and XPS. The properties of PtGd alloy thin films for resistive hydrogen sensing are investigated as a function of gas concentration, alloy composition and temperature.

## 2. Materials and Methods

The films were deposited onto 0.5 mm thick silicon substrates coated with 300 nm thermal oxide. The substrates were cut in the dimensions of 10 × 10 mm^2^ using a wafer dicer. Prior to alloy coating, the Si/SiO_2_ substrates were sequentially cleaned in acetone, isopropyl alcohol and washed in DI water using an ultrasonic bath for 10 min. We employed a co-sputtering deposition method using high-purity targets of Pt (3 inch diameter, 99.99% purity) and Gd (3 inch diameter, 99.97% purity) within a 6 target Nordiko 2000 (Nordiko, Hampshire, UK) magnetron sputtering system (deposition base pressure 5.5 × 10^−8^ Torr) to synthesize Pt_x_Gd_100′x_ ultrathin films with x = 25, 50 and 75. PtGd alloy films were fabricated using a precision-controlled deposition process, enabling deposition rates of 1.15 Å/s for Pt and 0.72 Å/s for Gd. To ensure uniform film thickness and consistent alloy composition, the sample holder was set to rotate under the two targets. The Dektak 3030ST profilometer (Bruker, Billerica, MA, USA) was used to calibrate the deposition rates of Gd and Pt, using thicker films. The fabrication involved multiple deposition cycles of submonolayers of Pt and Gd, with the total number of these cycles being adjusted to achieve the desired film thickness. Following the deposition, the samples were annealed at 300 °C for 2 h under vacuum conditions of 10^−2^ mbar to promote homogeneity across the film thickness and alloy formation. Characterization of these films through X-ray Photoelectron Spectroscopy (XPS) revealed notable shifts in the principal peaks of both Pt and Gd, indicative of effective alloy formation. The results confirm the efficacy of our methodological approach, combining low-rate deposition with annealing to achieve the targeted alloy structure characterized by high uniformity and compositional precision.

Structural characterization of the film was performed using scanning electron microscopy (SEM, LEO-EVO 40, Carl Zeiss, Oberkochen, Germany), Energy Dispersive X-ray spectroscopy (EDX, Bruker-125, Bruker, Billerica, MA, USA) and X-ray photoelectron spectroscopy (XPS, Specs-Flex) techniques. In the XPS setup, Al Kα was used as the X-ray source and the grazing angle was 55°. To fabricate a resistive sensor, silver contacts (dimensions: 5 × 2 mm^2^) separated by a distance of 1 mm are deposited on ultrathin layers of alloy PtGd films by thermal evaporation (PVD system-NONOVAK 400, Nanovak, Ankara, Türkiye). The electrical resistance of thin film sensors is consistently monitored in various environmental conditions using a custom-built measurement cell. Dry air serves as the carrier gas, with hydrogen concentration varying from 10 ppm to 5% (50,000 ppm), and the measurement temperature is controlled between 25 °C and 150 °C using a heater in the measuring cell. Detailed information about the measurement setup is provided in a previous paper [33].

## 3. Results

**Figure 1:**
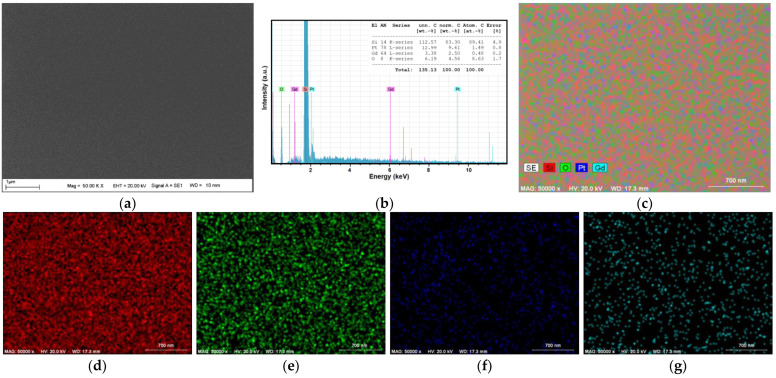
SEM image (**a**), EDX spectrum (**b**) and elemental mapping (full mapping (**c**), Si (**d**), O (**e**), Pt (**f**) and Gd (**g**)) of Pt_75_Gd_25_ alloy ultrathin film.

Figure 1a displays the SEM image of a Pt_75_Gd_25_ thin film, demonstrating a smooth surface without any segregation, and this observation remains consistent across other compositions of PtGd. Figure 1b exhibits the EDX spectrum of Pt_75_Gd_25_ ultrathin film, wherein the Pt and Gd peaks are prominently highlighted, while the remaining peaks originate from the Si/SiO_2_ substrate. The atomic percentage is calculated taking into account the atomic percentages of only Pt and Gd as seen in the upper-right corner of Figure 1b. Our EDX analysis revealed atomic percentage rates of 75.6% for Pt and 24.4% for Gd within the Pt_75_Gd_25_ alloy ultrathin films. Additionally, the EDX spectra of ultrathin PtGd alloy films, prepared with diverse chemical compositions, exhibited a remarkable agreement with our anticipated outcomes. The EDX elemental full, Si, O, Pt and Gd mapping are shown in Figure 1c–g, respectively. Bright red and green represent Si and O elements that are related to the substrate. Pt and Gd are seen all over the surface with green and turquoise in the form of lower brightness due to the ultrathin film.

**Figure 2:**
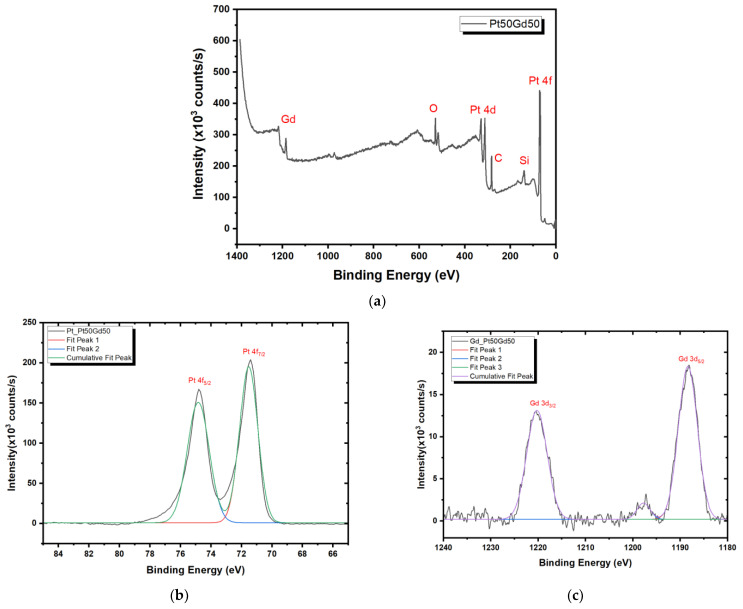
The full XPS spectra of the Pt_50_Gd_50_ thin film sample: (**a**) XPS spectra of the Pt_50_Gd_50_ alloy thin film, (**b**) Pt 4f region and (**c**) of Gd 3d region.

XPS was utilized in this study to analyze the PtGd alloy chemical composition. The XPS survey of the Pt_50_Gd_50_ thin film sample was performed (Figure 2a) and the spectra indicated the presence of Pt 4f (Figure 2b) and Gd 3d (Figure 2c) peaks. The elemental peaks of O and Si observed in the XPS analysis were attributed to the Si/SiO_2_ substrate. Furthermore, the elemental peaks of C can be attributed to surface impurities, indicating surface contamination. The Pt:Gd atomic ratio in the Pt_50_Gd_50_ alloy films were evaluated using XPS and high resolution XPS spectra looking for the Pt 4f and Gd 3d signature. The peak area of the Pt and Gd photoelectron signals were fit using a Gaussian profile, allowing the quantification of the chemical composition of the PtGd alloy films. The peak areas for Pt 4f and Gd 3d calculated by integration of the area under the Gaussian fitting curves were evaluated using the atomic sensitivity factor (ASF), specific to each element on the periodic table and customized for the XPS experimental setup, to analyze the chemical composition of the PtGd films. The calculated Pt and Gd ratio (Pt:Gd) based on layers of PtGd alloy in the sample were found to be 51:49. According to the detailed analysis of the XPS results, it is remarkable that the expected Pt 4f peak for platinum, which should appear at 71.2 eV, is instead observed at 71.6 eV. Upon examining the broad XPS spectrum of PtGd, peaks corresponding to elements such as C, Si and O are evident. Should Pt have formed an alloy with Pt(OH)_2_ or PtSi, the binding energy values for the Pt 4f peak would range between 72 and 73 eV [43]. Similarly, for gadolinium (Gd), while the expected binding energy for the Gd 3d peak in pure Gd is 1186 eV, our studies have recorded this peak at 1188 eV. In the broad spectrum, if Gd were bound with oxygen, forming a Gd_2_O_3_ structure, the Gd 3d peak position would be anticipated to lie between 1189 and 1190 eV [43]. Given these observations, we can conclude that an alloy formation between Gd and Pt has occurred. Previously, Peera and coworkers explained Pt 4f XPS peak shift with alloy formation, metal–support interaction and particle size for Pt-Y alloy in their review paper [44]. Also, there are various types of crystal structures such as orthorhombic, cubic, rhombohedral and so on depending on PtGd alloy composition [45]. In addition to this, the shape of Pt and Gd peaks have different forms than their pure elemental peaks [43]. These results provide further evidence for the successful fabrication of the PtGd alloy thin film.

**Figure 3:**
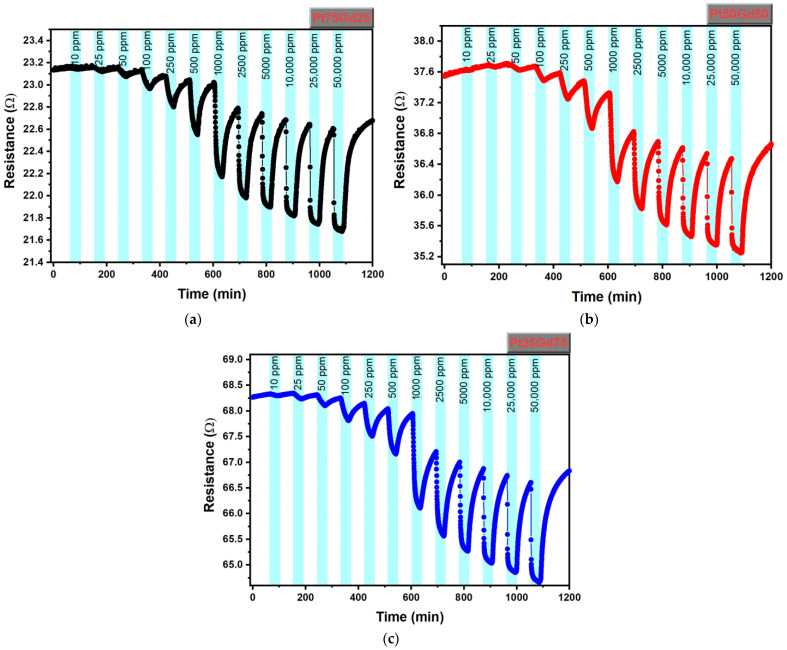
The resistance over time for (**a**) Pt_75_Gd_25_, (**b**) Pt_50_Gd_50_ and (**c**) Pt_25_Gd_75_ thin film samples, upon injection of a certain hydrogen concentration and air at the temperature of 150 °C.

The resistive hydrogen sensing properties of PtGd alloy thin films is investigated depending on temperature, concentration and Gd content in the alloy. Figure 3a–c show the resistance as a function time for Pt_75_Gd_25_, Pt_50_Gd_50_ and Pt_25_Gd_75_ thin film samples during exposure to various hydrogen concentrations from 10 ppm to 50,000 ppm at 150 °C, respectively. The resistances of all Pt_75_Gd_25_, Pt_50_Gd_50_ and Pt_25_Gd_75_ thin film sensors decreases when hydrogen is introduced into the measurement cell at the specified concentration and increases when the measurement cell is purged with dry air. The hydrogen gas exposure time at a certain concentration was kept constant at 30 min and the dry air cleaning time was kept constant at 45 min. Surface scattering phenomenon may contribute to reduced Pt nanostructure resistance in hydrogen environments, as referenced in studies [18,19,20,21,24]. When oxygen atoms from dry air are absorbed, they coat the film’s surface. Introducing hydrogen into this environment causes hydrogen atoms to displace oxygen atoms, reducing electron scattering at the film surface. Consequently, this leads to a decrease in the film’s resistance.

The determination of sensor response, a fundamental parameter within the context of resistive gas sensors, can be accomplished through the utilization of the subsequent equation

(1) $$\mathrm{Sensor}\;\mathrm{response}\;\left(\%\right)=\frac{∆R}{R_{H}}\times 100=\frac{R_{0}-R_{H}}{R_{H}}\times 100$$

where Δ*R* signifies the alteration in the resistance exhibited by the resistive sensor apparatus, $R_{0}$ pertains to the baseline resistance value that defines the sensor’s response when subjected to an environment of controlled dry air and $R_{H}$ signifies the resistance manifested by the sensor after its exposure to a designated concentration of hydrogen.

**Figure 4:**
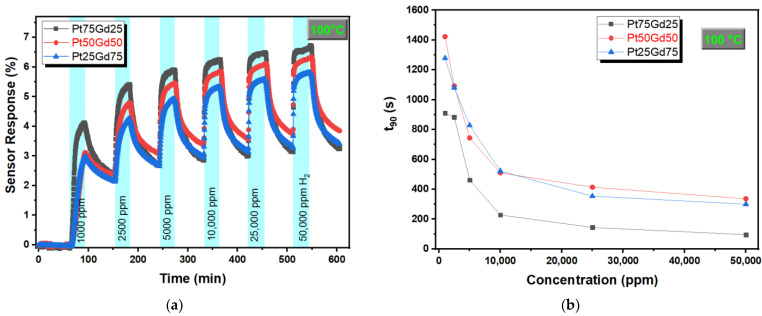
(**a**) Sensor response versus time and (**b**) response time as a function of concentration for PtGd alloy thin films at 100 °C during exposure to different hydrogen concentrations from 1000 ppm to 50,000 ppm.

Figure 4a shows the sensor response versus time for PtGd alloy thin films at 100 °C during exposure to different hydrogen concentrations from 1000 ppm to 50,000 ppm. The highest sensor response is observed for the Pt_75_Gd_25_ alloy thin film sensor and there is no dramatical decrease with an increase in Gd content in the alloy. The sensor responses of Pt_75_Gd_25_, Pt_50_Gd_50_ and Pt_25_Gd_75_ alloy thin film sensors are observed as approximately 4.1, 3.1 and 2.95 for exposure to 1000 ppm hydrogen at 100 °C and these values were higher than the sensor response of the bare Pt thin film sensor of about 2.4 at the same temperature and hydrogen concentration [24,32]. The sensor response enhances with an increase in hydrogen concentration, as seen in Figure 4a. Response time (t_90_) could be defined as the time required for the resistance of the alloy thin film to reach 90% of the total change in the resistance when exposed to the indicated hydrogen concentration. Figure 4b shows the t_90_ as a function of hydrogen concentration for all PtGd alloy thin films at 100 °C. The response time decreases and then the decrease rate slows down with an increase in hydrogen concentration for PtGd alloy thin films. This behavior was reported for various nanostructure types of Pt and could be related to a long time requirement to be fully adsorbed onto the thin film surface [18,19,29,32]. On the other hand, as expected, the response time decreases with an increase in temperature due to a higher diffusion of hydrogen at high temperatures. The minimum response time is obtained for the Pt_75_Gd_25_ alloy thin film for all measured hydrogen concentrations as seen in Figure 4b. The response time of the Pt_75_Gd_25_ alloy thin film for 1000 ppm hydrogen concentration is about 910 s and this value is higher than the response time of the diverse bare Pt nanostructures thin film and nanowire at the same condition. One reason for this high response time could be the high measurement cell volume (1 L) and the high dead volume of our system by comparing to the 200 sccm flow rate

**Figure 5:**
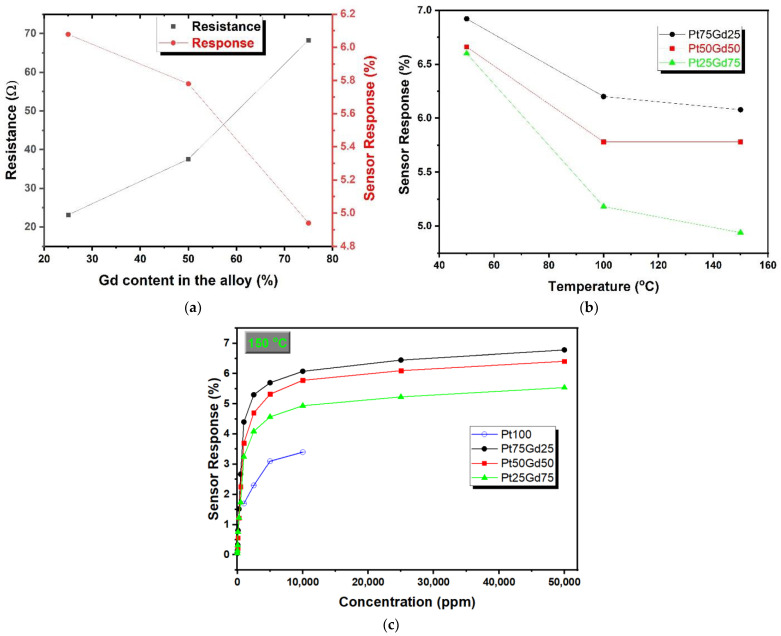
(**a**) The sensor response of PtGd alloy films exposed to 10,000 ppm hydrogen and the resistance under dry air versus Gd content in the alloy at 150 °C. (**b**) The sensor response of PtGd alloy thin films as a function of temperature during exposure to 10,000 ppm hydrogen. (**c**) The sensor responses of PtGd alloy thin films at 150 °C as a function of hydrogen concentration. The result of pure Pt was added from [24].

Figure 5a displays the resistance under dry air and sensor response of PtGd alloy films exposed to 10,000 ppm hydrogen as a function of Gd content in the alloy at 150 °C. The resistance of PtGd alloy thin films enhances from approximately 23.1 Ω for the Pt_75_Gd_25_ thin film sample to 68.2 Ω for the Pt_25_Gd_75_ thin film sample with increasing Gd content in the alloy. In a broader context, encompassing the study of alloy resistivity behavior, a comprehensive understanding can be attained by invoking the following two established principles: Matthiessen’s rule, which pertains to dilute alloys characterized by a foreign element content constituting less than 5% of the alloy’s atomic composition, and Nordheim’s rule, which is applicable to concentrated alloys [46].

Focusing specifically on the present scenario, we embarked on the synthesis of a concentrated PtGd alloy. In this context, our investigation of the alloy’s electrical properties has revealed a consistent alignment with the fundamental tenets of Nordheim’s rule. Within the framework of Nordheim’s rule, the calculation of resistivity can be accomplished through the utilization of the subsequent equation [46]

(2) $$\rho =A+c\left(1-Bc\right)$$

where *A* and *B* constitute constants that are intricately linked to the alloy series and *c* signifies the concentration of a specific component embedded within the alloy’s composition. Prior research conducted by Blood and Greig demonstrated a discernible trend in the context of concentration-dependent resistivity for PtPd alloys, revealing a graph characterized by a roughly parabolic shape [47]. In a similar vein, Litschel and Pop undertook an investigation of the electrical resistivity of Ni_1−x_Pt_x_ alloys, with the parameter “x” representing a compositional range spanning from 0 to 30 atomic percent. This research was conducted in relation to varying temperature conditions. Remarkably, their findings unveiled distinct resistivity profiles for different compositions as follows: the resistivity of pure Ni was measured at 7.0 × 10^−6^ Ω.cm, while for Ni_0.7_Pt_0.3_, it was notably higher at 33.3 × 10^−6^ Ω.cm [48].

The sensor response of PtGd alloy thin films exposed to 10,000 ppm hydrogen decreases from approximately 6.1 for the Pt_75_Gd_25_ sample to 4.9 for the Pt_25_Gd_75_ sample with increasing Gd content in the alloy as seen in Figure 5a. The sensor response of PtGd alloy thin films as a function of the temperature graph during exposure to 10,000 ppm hydrogen is presented in Figure 5b. Sensor responses of all PtGd alloy thin film samples decrease with an increase in temperature. Similarly, a decrease in the sensitivity with enhancing temperature were observed for the Pt-modified Pd nanowire, Pt thin film and nanoporous Pt film [13,24,29]. Figure 5c gives the sensor response as a function of hydrogen concentration for PtGd alloy thin films at 150 °C and the sensor response enhances rapidly and slowly in low and high hydrogen concentrations, respectively. In order to compare PtGd alloy and pure Pt thin film sensors, the results for pure Pt thin film from the previous paper [24] are seen in Figure 5c. PtGd alloy film sensors show better sensor response than the response of pure Pt thin film for the indicated hydrogen concentration range. The increased sensor response of PtGd alloy thin film resistive hydrogen sensors could be related to the increase in the lattice parameter, the modifications in the electronic band structure, the changes in the crystal structure and the formation of hydrides. The atomic radius of Pt is 2.3 Å, while that of Gd is 2.77 Å [49]. During the formation of the PtGd alloy, Gd atoms are replaced with Pt atoms and, as expected, the lattice parameter increases compared to pure Pt [41]. Also, the PtGd alloy exhibits various crystal structures depending on the alloy ratio [45]. Therefore, compared to pure Pt, an expanded lattice parameter could facilitate hydrogen diffusion not only on the surface but also into the film, potentially forming metal hydride. This could explain the high sensitivity of the PtGd alloy and why hydrogen desorption from both the surface and within the film takes longer. The Henry’s isotherm model, the simplest absorption isotherm, is not suitable for our case due to the nonlinearity between sensor response and hydrogen concentration. Henry’s adsorption isotherm illustrates a linear relationship between the amount of analyte adsorbed and its equilibrium concentration, as shown in Equation (3) [50]. The sensor response should be related and proportional to the number of the adsorbed analyte

(3) $$\mathrm{Sensor}\;\mathrm{Response}\;\approx q_{e}=K_{H}C_{e}$$

where $q_{e}$ is the adsorbed analyte amount at equilibrium, $K_{H}$ is Henry’s adsorption constant and $C_{e}$ is the adsorbate’s equilibrium concentration.

**Figure 6:**
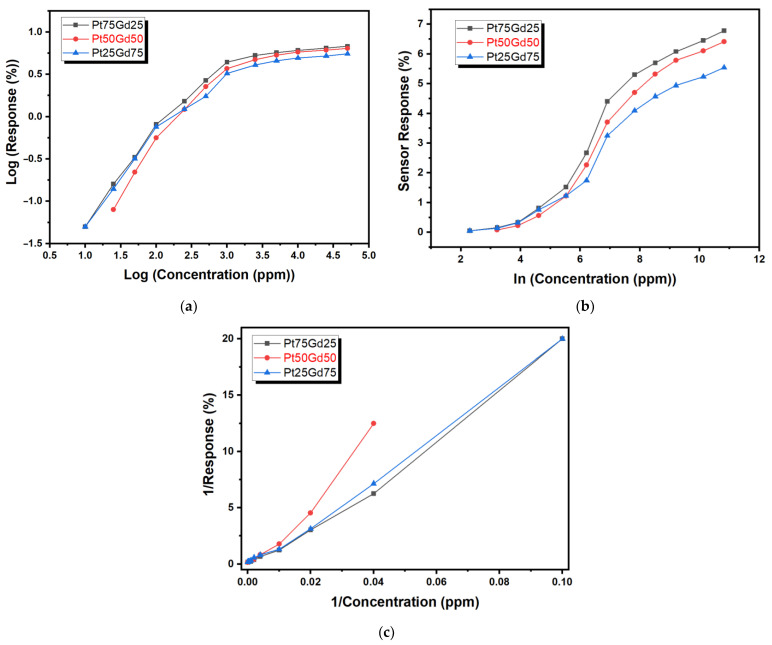
Sensor response as a function of hydrogen concentration for PtGd alloy thin films at 150 °C in different mathematical models: (**a**) log (sensor response (%)) versus log (concentration (ppm)) graph, (**b**) sensor response versus ln (concentration (ppm)) graph and (**c**) 1/Response versus 1/Concentration (ppm) graph.

Freundlich’s absorption isotherm proposed for multilayer adsorption and applied for heterogeneous surface sites over a small concentration range [51,52]. The mathematical model can be given as

(4) $$\mathrm{Sensor}\;\mathrm{Response}\;\approx q_{e}=K_{F}{C_{e}}^{1/n}$$

where $K_{F}$ is the absorption capacity coefficient and 1/n is the coefficient of surface heterogeneity. If the value of 1/n is between 0 and 1, the adsorption process is favorable. If the value of 1/n is grater than 1, the adsorption process is unfavorable. If the value of 1/n is equal to 1, the adsorption process is irreversible. Figure 6a shows a log (sensor response) versus log (concentration) graph for PtGd alloy thin films at 150 °C. Freundlich’s absorption isotherm model is not suitable for PtGd thin films resistive hydrogen sensors.

Temkin’s absorption isotherm ignores extreme high and low concentration values and is based on the multilayered chemisorption process [51,53]. Temkin’s absorption isotherm model is expressed as

(5) $$\mathrm{Sensor}\;\mathrm{Response}\;\approx q_{e}=\frac{RT}{b_{T}}\ln\left(A_{T}C_{e}\right)$$

where R is the universal gas constant, T is the temperature in Kelvin, $b_{T}$ is the Temkin constant related to sorption heat and $A_{T}$ is the Temkin isotherm constant. Figure 6b shows sensor response as a function of the concentration (natural logarithm scale) for PtGd alloy thin films at 150 °C and the relationship between the sensor response and ln (concentration) is not linear. The coefficients of determination (R^2^) are 0.95, 0.96 and 0.95 for Pt_75_Gd_25_, Pt_75_Gd_25_ and Pt_25_Gd_75_ alloy thin film sensors, respectively. Therefore, Temkin’s absorption isotherm model is not suitable for PtGd thin films resistive hydrogen sensors.

Langmuir’s absorption isotherm is based on the single-layer absorption process that assumes monolayer adsorption, constant adsorption energy, homogeneous sites and no interaction between the adsorbed molecules [51,54]. A nonlinear matemathical form of Langmuir’s absorption isotherm model is given as

(6) $$\mathrm{Sensor}\;\mathrm{Response}\;\approx q_{e}=\frac{Q_{o}K_{L}C_{e}}{1+K_{L}C_{e}}$$

where $K_{L}$ is the Langmuir constant and $Q_{o}$ is the maximum amount of adsorbed analyte. Figure 6c shows the dependency of the sensor response with the concentration for PtGd alloy thin films at 150 °C. The coefficients of determination (R^2^) of Pt_75_Gd_25_, Pt_75_Gd_25_ and Pt_25_Gd_75_ alloy thin film sensors are 0.99, 0.97 and 0.995, respectively. Langmuir’s absorption isotherm model is the best fit for our data for PtGd thin films resistive hydrogen sensors. The noise of the resistances of the sensor devices for 10 min under dry air flow and fitting the 1/response versus 1/concentration plot in Figure 6c are considered for the calculation of the limit of detection (LOD). The LOD values of Pt_75_Gd_25_, Pt_75_Gd_25_ and Pt_25_Gd_75_ alloy thin film sensors are 4 ppm, 15 ppm and 3 ppm, respectively.

## 4. Discussion and Conclusions

Table 1 summarizes reported data on the sensor response of Pt thin films–based resistive hydrogen sensors for different thicknesses, chemical compositions and different structural forms. The findings show that PtPd bimetallic films exhibit the highest sensitivity results at 150 °C with 1% hydrogen concentration, compared to other reported systems. Additionally, Pt films of 5 nm at 200 °C showed high sensitivity values when exposed to hydrogen concentrations of 500 ppm.

In this paper, we made a contribution to the field of hydrogen gas detection by measuring the sensor response of PtGd alloy samples, which has not been previously reported in the literature. The PtGd alloy films were exposed to 10,000 ppm of hydrogen at different temperatures, and the resulting sensitivity factors were obtained as 6.1 (Pt_75_Gd_25_), 5.8 (Pt_50_Gd_50_) and 4.9 (Pt_25_Gd_75_) at the temperature of 150 °C. These findings suggest that PtGd alloy films have potential as highly sensitive materials for hydrogen gas detection, especially in situations that require a high level of sensitivity. The results also highlight the potential of PtGd alloy as a material for improving the selectivity of hydrogen gas sensors, which is a critical factor for reliable and accurate detection in real-world applications. Furthermore, this study provides insights into the effects of varying the composition of the PtGd alloy films on their sensitivity and selectivity toward hydrogen gas. The temperature-dependent electrical properties of the PtGd alloys were evaluated using Nordheim’s rule, and our findings exhibited a strong correlation. In addition to studying the electrical characteristics, we explored the absorbtion isotherm characteristics of the PtGd films through several renowned models, including Henry, Temkin, Freundlich and Langmuir absorption isotherms. Among these models, the Langmuir’s absorption isotherm demonstrated the closest alignment with the outcomes of PtGd thin film resistive hydrogen sensors. This information could be useful for the design and optimization of hydrogen gas sensors with improved performance characteristics. Overall, our results demonstrate the potential of PtGd alloy films as a new class of materials for hydrogen gas detection, which could have significant implications for a wide range of applications, including hydrogen fuel cells, chemical processing and environmental monitoring.

## Figures and Tables

**Table 1:** Comparison of the hydrogen sensor response across various Pt-based materials in thin film forms.

Thin Films	H_2_ Conc. (ppm)	Temperature (°C)	Sensor Response (%)	LOD (ppm)	Ref.
		RT	1		
3.5 nm Pt	500	100	~4	10 *	[20]
		200	~8		
10 nm Pt			~1.5		
20 nm Pt	10,000	RT	~0.5	10 *	[22]
40 nm Pt			~0.4		
5 nm Pt	10 000	RT	~4	100 *	[55]
2 nm Pt	1000	RT	~2.8	1000 *	[24]
5 nm Pt	200	150	~4.5	1	[21]
2 nm Pt	1000	RT	~2.4	<1	[23]
150 nm nanoporous Pt	1000	RT	3.5	<100	[13]
3 nm nanoporous Pt	10,000	RT	13.0	15	[14]
PtPd bimetallic	10,000	150	13.5	10 *	[25]
2 nm Pt_0.75_Co_0.25_ alloy	10,000	150	1.25	1000 *	[33]
2 nm Pt_79_Ni_21_ alloy	1000	RT	~2.6	<1	[32]
2 nm Pt_75_Gd_25_ alloy			6.1	4	
2 nm Pt_50_Gd_50_ alloy	10,000	150	5.8	15	This work
2 nm Pt_75_Gd_25_ alloy			4.9	3	

* No LOD value was given in the paper; this value is the lowest concentration measured.

## Data Availability

The data that support the findings of this study are available from the corresponding author upon reasonable request.
